# Physiology of Conception

**Published:** 1842-03

**Authors:** 


*Physiology of Conception.*—We find the following interesting case in the November number of the *Archives Generales de Medicine*, where it is credited to Riecke's Prussian Medical Gazette.

A girl aged twenty eight, servant in the family of a peasant, had for some time been suspected of a criminal intimacy with her master—her mistress at length surprised them in *flagrante delicto* and turned the girl away—two days after this the dead body of the unfortunate girl was found in a neighboring pond, an autopsy was ordered by the magistrate, and the organs of generation were found in the following condition:

The labia majora were a little tumified, the hymen very much stretched and recently torn, much mucus in the vagina. The bladder, rectum, and other pelvic organs, were very much injected, the uterus was of a deep red color and more than one third larger than in the normal state. The cavity of the organ which was also increased in size was filled with a sanguinolent mucus, of which that portion immediately in contact with the walls of the uterus was most consistent. The lining membrane was red and soft. The right fallopian tube was red, and its size about three times greater than in the normal condition; it was swollen in the middle. A pretty large sound (*un sonde assez epaisse*) penetrated the canal with ease and encountered no obstacle till it reached the uterine extremity of the tube where the swelling existed; the cavity of the tube was filled with a white fluid. The right ovary was double the size of the other, it was embraced by the fimbriæ of the fallopian tube and appeared very much injected.

We observed upon its surface two chinks of the size of a lentil, and very red at the bottom. From each of these issued a delicate membrane half an inch long, and bearing at its extremity a small ovule (petite œuf) about the size of a grain of mustard seed. Each of these ovules presented, even to the naked eye, upon their surfaces, a net work of vessels very distinct indeed; they contained a transparent fluid. The ovary having been laid open, we found within it a red coagulum granular towards the centre; this coagulum communicated with the chinks before mentioned.

From these facts the presumption was, that conception took place two days before, but it was impossible to arrive at any thing like certainty.

*Remarks.*—This case is one of the most valuable yet recorded in the illustration of the physiology of conception. It is exceedingly to be regretted that no microscopic examination of the ovule was made; some of the vexed questions of obstetrics might possibly have been settled, had these minute ovules been subjected to accurate examination—particularly as to whether the ovule has two coats, as obstetric writers generally have taught, or three as Von Baer asserts, or only one as some of the late physiologists pretend, the chorion, according to their view, being a capsule added to the ovum during its transit through the Fallopian tube. The excessive vascularly observed in this case in all the pelvic organs and especially in the uterus is very interesting; how well it illustrates the remark of Breschet, that the formative process, like the reparative, partakes largely of the character of inflammation. The state of the sanguinolent mucus by which the uterus was occupied, fluid in the centre and becoming of more solid consistence upon the uterine wall, shows us very distinctly how the decidua is formed, and why it happens that this shut sac when fully developed should contain a fluid in its cavity. The sanguinolent mucus is separated into two parts, a soft solid immediately applied upon the uterine wall and there forming the decidua (perione of Breschet,) and a fluid, the hydro-perione of Breschet, occupying the cavity of the membrane.—*N. Y. Med. Gaz.*

